# Ochracenoids A and B, Guaiazulene-Based Analogues from Gorgonian *Anthogorgia ochracea* Collected from the South China Sea

**DOI:** 10.3390/md12031569

**Published:** 2014-03-14

**Authors:** Juan-Juan Zheng, Chang-Lun Shao, Min Chen, Li-She Gan, Yu-Chun Fang, Xu-Hui Wang, Chang-Yun Wang

**Affiliations:** 1 Key Laboratory of Marine Drugs, Ministry of Education, School of Medicine and Pharmacy, Ocean University of China, Qingdao 266003, China; E-Mails: zhengjuanjuan90@gmail.com (J.-J.Z.); shaochanglun@ouc.edu.cn (C.-L.S.); carbohydrateyu@gmail.com (M.C.); yuchunfang@ouc.edu.cn (Y.-C.F.); haiyangbencao@ouc.edu.cn (X.-H.W.); 2 Institute of Modern Chinese Medicine, College of Pharmaceutical Sciences, Zhejiang University, Hangzhou 310058, China; E-Mail: lsgan@zju.edu.cn

**Keywords:** gorgonian, *Anthogorgia ochracea*, guaiazulene-based analogue, antiproliferative effect, zebrafish embryo

## Abstract

Two new guaiazulene-based analogues, ochracenoids A (**1**) and B (**2**), along with four known analogues (**3**–**6**), were isolated from the gorgonian *Anthogorgia ochracea* collected from the South China Sea. The planar structures of the new compounds were elucidated by comprehensive spectroscopic data. The absolute configuration of **1** was determined as 3*R* by the comparison of TDDFT calculated electronic circular dichroism with its experimental spectrum. Compound **1** is a rare guaiazulene-based analogue possessing a unique C_16_ skeleton. The possible generation process of **1** through an intermolecular one-carbon-transfer reaction was also discussed. Compound **2** was previously described as a presumed intermediate involved in the biogenesis of anthogorgienes A and I. Compound **3** exhibited antiproliferative effects on the embryo development of zebrafish *Danio rerio*.

## 1. Introduction

**Figure 1:**
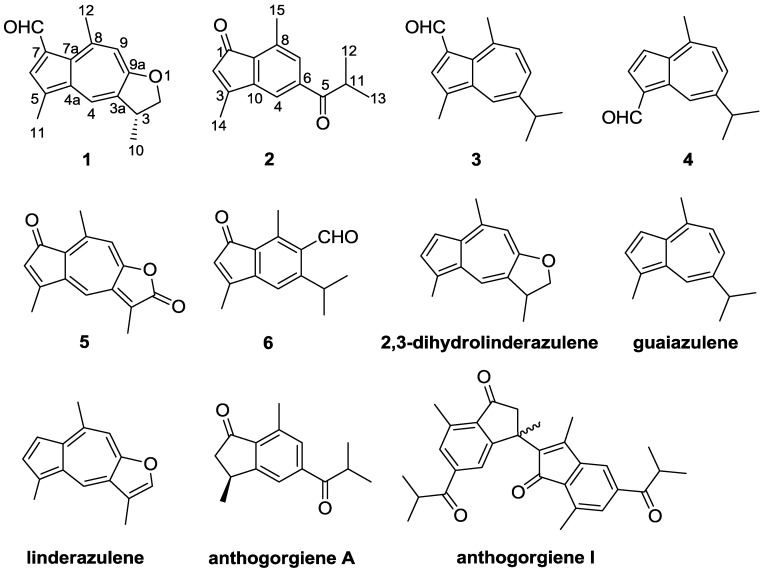
Structures of compounds **1**–**6** and related compounds.

Gorgonians have proven to be a rich source of guaiazulene-related pigments [1]. Guaiazulene-based analogues are well recognized for their distinctive blue and purple colors, which are a part of the origin of the brilliant colors of gorgonians and other organisms [2]. These compounds feature an azulene core, similar to the fused five-seven bicyclic aromatic ring system [3]. They have received much attention due to their multiple potent biological activities including antifungal, antitumor, and immunoregulatory activities and antiproliferative effects on fertilized sea urchin eggs [4,5]. In particular, a series of guaiazulene-based compounds were reported from gorgonians of *Anthogorgia* [2,6], and *Acalycigorgia* (synonymous with *Anthogorgia*) [4,7]. Recently, in the course of our investigation on new bioactive substances from gorgonians and soft corals as well as their derived fungi from the South China Sea [8,9,10], the gorgonian *Anthogorgia ochracea* collected from the South China Sea attracted our attention because the EtOAc extract of the gorgonian showed the presence of guaiazulene-based sesquiterpenes with characteristic UV absorption spectra (UVA λ_max_ 320–400 nm) [11]. Chemical investigation on the extract led to the isolation of two new guaiazulene-based analogues, ochracenoids A (**1**) and B (**2**), and four known related analogues (**3**–**6**) (Figure 1). Herein, we report the isolation, structure elucidation, and biological activities of these compounds. As these compounds have been found to be quite labile and easily decomposable on exposure to air and light during the work-up [12], the possible generation process of **1** was also discussed.

## 2. Results and Discussion

**Figure 2:**
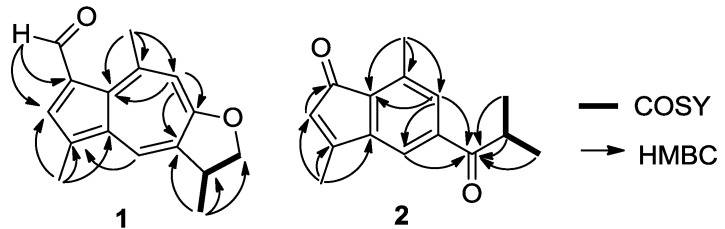
^1^H-^1^H COSY and key HMBC correlations for **1** and **2**.

Ochracenoid A (**1**) was obtained as a red-purple solid. Its molecular formula of C_16_H_16_O_2_ (9 degrees of unsaturation) was determined by HRESIMS. The IR spectrum of **1** showed an absorption band corresponding to a formyl functionality (1655 cm^–1^). The ^13^C NMR (Table 1) spectrum indicated the presence of 16 carbon resonances including a formyl carbon (*δ*_C_ 187.0), seven quaternary olefinic carbons (*δ*_C_ 166.7, 149.0, 138.5, 134.7, 130.3, 129.2 and 127.6), three olefinic methines (*δ*_C_ 135.6, 131.0 and 116.0), one methine (*δ*_C_ 39.4), one methylene (*δ*_C_ 78.1), and three methyl groups (*δ*_C_ 30.9, 20.5 and 13.1). The ^1^H NMR (Table 1) spectrum showed signals corresponding to two aromatic methyls [*δ*_H_ 3.11 (s, 3H) and 2.55 (s, 3H)] and three aromatic methines [*δ*_H_ 8.12 (d, *J* = 1.2 Hz, 1H), 7.97 (s, 1H) and 7.09 (s, 1H)]. These spectroscopic data indicated that the basic skeleton of **1** should be an azulene, and the ^1^H NMR signals for the azulene portion of **1** were similar to those of linderazulene (Figure 1) [13]. The remaining resonances, one methyl at *δ*_H_ 1.49 (d, *J* = 6.6 Hz), one methine at *δ*_H_ 3.81 (m), and one methylene at *δ*_H_ 4.82 (t, *J* = 8.4 Hz) and 4.27 (dd, *J* = 8.4, 6.6 Hz), suggested that **1** was a 2,3-dihydro-derivative of linderazulene. The NMR spectra of **1** were nearly identical to those observed for 2,3-dihydrolinderazulene (Figure 1) [4], a guaiazulene-based analogue isolated from an *Anthogorgia* gorgonian. The main difference was the observation of resonances attributable to a formyl functionality [*δ*_C_ 187.0 (CH) and *δ*_H_ 10.61 (s, 1H)] connected to C-7 in **1**, supported by the HMBC correlations from –CHO to C-7 and C-6 (Figure 2), rather than an olefinic proton in 2,3-dihydrolinderazulene. The planar structure of **1** was confirmed by 2D-NMR experiments including ^1^H-^1^H COSY, HMQC, and HMBC correlations (Figure 2). Compound **1** was therefore designated as 7-formyl-2,3-dihydrolinderazulene.

**Table 1:** NMR spectroscopic data (600/150 MHz, CDCl_3_) for compound **1**.

Position	*δ*_H_ (*J* in Hz)	*δ*_C_ Type	HMBC
2	4.82, t (8.4)	78.1, CH_2_	C-10
	4.27, dd (8.4, 6.6)		
3	3.81, m	39.4, CH	-
3a	-	130.3, C	-
4	8.12, d (1.2)	131.0, CH	C-3, C-5, C-7a, C-9a
4a	-	138.5, C	-
5	-	127.6, C	-
6	7.97, s	135.6, CH	C-4a, C-7a
7	-	129.2, C	-
7a	-	134.7, C	-
8	-	149.0, C	-
9	7.09, s	116.0, CH	C-3a, C-7a, C-9a, C-12
9a	-	166.7, C	-
10	1.49, d (6.6)	20.5, CH_3_	C-2, C-3, C-3a
11	2.55, s	13.1, CH_3_	C-4a, C-5, C-6
12	3.11, s	30.9, CH_3_	C-7a, C-8, C-9
7-CHO	10.61, s	187.0, CH	C-6, C-7

**Figure 3:**
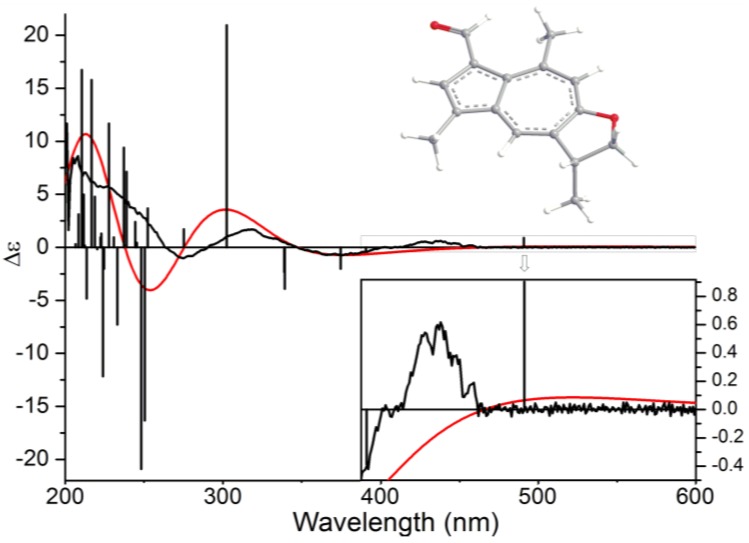
B3LYP/6-311++G(2d,2p)/B3LYP/6-31+G(d) calculated electronic circular dichroism (ECD) spectrum of 3*R*-**1** (*red*) and the experimental ECD spectrum of **1** (*black*).

The absolute configuration of C-3 in **1** was determined by theoretical calculation of electronic circular dichroism (ECD) spectrum. Arbitrarily assigned 3*R*-**1** was employed to perform a random conformational search by using the Monte Carlo method with MMFF94 force field in the Spartan 08 software package [14]. Only one lowest-energy conformer has been located within an energy cut-off of 2 kcal/mol (Figure 3). Full geometry optimization and harmonic vibrational frequencies calculation were performed at the B3LYP/6-31+G(d) level in the gas phase by using the Gaussian 09 software package [15]. The energies, oscillator strengths, and rotational strengths of the first 30 electronic excitations were calculated using the TDDFT methodology at the B3LYP/6-311++G (2d,2p)/B3LYP/6-31+G(d) level in vacuum. The ECD spectra were then simulated by the overlapping Gaussian function (*σ* = 0.5 eV) [16]. In the 200–600 nm region, compared to the experimental ECD spectrum of **1**, the calculated ECD spectrum of 3*R*-**1** showed a similar curve with positive first, negative second, positive third, and negative fourth Cotton effects at 520 (+82), 375 (−1), 303 (+15), and 254 (−20) nm, respectively (Figure 3). Although there were some large wavelength differences between experimental Cotton effects and the corresponding theoretical ones, especially the first Cotton effect, due to idealized modeling and simulation, qualitative analyses of the results allowed the assignments of the experimental first positive Cotton effect around 438 nm to the predicted first positive electronic excitation at 491 nm, the experimental second negative Cotton effect around 376 nm to the predicted 2nd–5th negative electronic excitations at 391, 375, 339, and 339 nm, and so on (Figure 3). Base on the above assignments, the absolute configuration at C-3 in **1** was determined as *R*.

Ochracenoid B (**2**) was obtained as a yellow solid, with a molecular formula of C_15_H_16_O_2_ (8 degrees of unsaturation) determined by HRESIMS. The ^1^H NMR spectrum (Table 2) indicated the presence of three aromatic methines [*δ*_H_ 7.61 (brs, 1H), 7.48 (brs, 1H) and 5.76 (s, 1H)], one methine [*δ*_H_ 3.52 (septet, *J* = 8.5 Hz, 1H)], and four methyls [*δ*_H_ 2.58 (s, 3H), 2.27 (s, 3H) and 1.23 (d, *J* = 8.5 Hz, 6H)]. The ^13^C NMR and DEPT spectra showed 15 carbon signals, including two ketones (*δ*_C_ 204.2 and 198.3), five quaternary olefinic carbons (*δ*_C_ 161.1, 146.7, 139.9, 136.6 and 131.2), three olefinic methines (*δ*_C_ 133.4, 125.2 and 116.5), one methine (*δ*_C_ 35.9), and four methyl groups (*δ*_C_ 19.2, 19.2, 17.3 and 14.2). The HMQC spectrum assigned all protonated carbons. These spectroscopic features suggested that **2** was structurally related to anthogorgiene A (Figure 1) [2]. The evident difference was that two sp^2^ olefinic carbons attributed to C-2 and C-3 presented in **2** instead of two sp^3^ carbons corresponding in anthogorgiene A. On the basis of 2D NMR analyses (Figure 2), an indenone nucleus was established. The methyl substitutions at C-3 and C-8 were confirmed by HMBC correlations from H_3_-14 to C-2, C-3 and C-10, and from H_3_-15 to C-7, C-8 and C-9, respectively. The presence of an isobutanoyl group was recognized from the ^1^H-^1^H COSY correlations between a methine proton (*δ*_H_ 3.52) and two methyl protons (*δ*_H_ 1.23), together with the HMBC interactions from the methyl and methine protons to the ketone (*δ*_C_ 204.2, C-5). The linkage of the isobutanoyl group to C-6 was deduced by the HMBC correlations from H-4 and H-7 to C-5. Thus the structure of **2** was established as 3,8-dimethyl-6-isobutanoylindenone.

**Table 2:** NMR Spectroscopic Data (500/125 MHz, CDCl_3_) for compound **2**.

Position	*δ*_H_ (*J* in Hz)	*δ*_C_ Type	HMBC
1	-	198.3, C	-
2	5.76, s	125.2, CH	C-1, C-3, C-9, C-10, C-14
3	-	146.7, C	-
4	7.48, brs	116.5, CH	C-5, C-7, C-9, C-10
5	-	204.2, C	-
6	-	139.9, C	-
7	7.61, brs	133.4, CH	C-4, C-5, C-9, C-15
8	-	136.6, C	-
9	-	131.2, C	-
10	-	161.1, C	-
11	3.52, septet (8.5)	35.9, CH	C-5, C-12, C-13
12	1.23, d (8.5)	19.2, CH_3_	C-5, C-11, C-13
13	1.23, d (8.5)	19.2, CH_3_	C-5, C-11, C-12
14	2.27, s	14.2, CH_3_	C-2, C-3, C-10
15	2.58, s	17.3, CH_3_	C-7, C-8, C-9

The structures of known compounds, 1-formylguaiazulene (**3**) [17], 1-formyl-4-methyl-7-isopropylazulene (**4**) [18], ketolactone (**5**) [19], and 3,8-dimethyl-5-isopropyl-6-formylindenone (**6**) [20] were elucidated by NMR spectroscopic data and comparison with those previously reported in the literature.

**Scheme 1:**
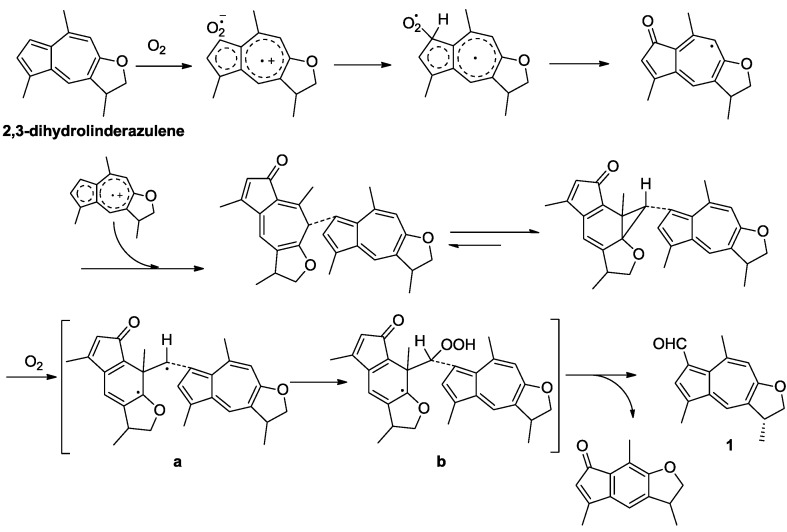
Possible transformation process from 2,3-dihydrolinderazulene to **1**.

A literature survey revealed that guaiazulene and related compounds could gradually suffer autoxidation even on standing at room temperature to give various products [21]. In the present study, guaiazulene-based analogues, including two C_16_-guaiazulene analogues (**1** and **3**), two indenone derivatives (**2** and **6**), and two common guaiazulenes (**4** and **5**), were obtained simultaneously. Compound **5** was reported to be a photo-oxidation product derived from linderazulene [22]. Compounds **3**, **4**, and **6** were described to be generated from the same precursor guaiazulene (Figure 1) and followed by an intermolecular one-carbon-transfer reaction, a side-chain oxidation, and a rearrangement to indenone derivatives, respectively [23]. Interestingly, guaiazulene-based sesquiterpenes commonly have fifteen carbon atoms, while **1**, as well as **3**, contained a unique C_16_ skeleton. Based on the fact that **1** is constructed by a guaiazulene sesquiterpene moiety, 2,3-dihydrolinderazulene, with an additional formyl attached at C-7, a possible oxidative transformation process from 2,3-dihydrolinderazulene to **1** was proposed in Scheme 1. By the attack of oxygen, 2,3-dihydrolinderazulene was considered to initially form an electron-transfer complex, followed by the conformation of two tautomeric dimers in equilibrium [2,21,23]. Then the unique tautomers constituted the important intermediates (Scheme 1, **a** and **b**) by exposure to air at room temperature. Finally, the intermediates were converted into **1** by an intermolecular one-carbon-transfer reaction to form the additional formyl group [21,23]. In the literature, **2** was described as a presumed intermediate involved in the biogenesis of anthogorgienes A and I (Figure 1), and was depicted to be derived from guaiazulene via a C-1 and C-5 peroxidated intermediate to follow a 4,5,6-cyclopropane formation and then cleavage of the C-4/C-5 bond [2]. The isolation of **2** gave evidence to the deduction of the generation process from guaiazulene to anthogorgienes A and I.

All the isolated compounds were evaluated for their antiproliferative effects on zebrafish embryo. It should be pointed out that the developing zebrafish *Danio rerio* is an *in vivo* pharmacologically relevant model which provides rapid and high throughput screening (HTS) for compounds with capability of reducing cell proliferation [24]. Compound **3** showed strong antiproliferative effects leading to abnormal several aspects of the embryonic development including coagulated eggs (48 h), notochord malformation (72 h), and embryo death (72 h) with the EC_50_ values of 3.98, 6.50, and 7.39 μM, respectively, while compounds **1**, **72** and **4**–**6** exhibited no antiproliferative effects on zebrafish embryo.

The isolated compounds were also evaluated for their cytotoxicities and antibacterial activities. However, no compound showed any cytotoxicity against five human tumor cell lines (HeLa, A-549, HCT-116, HL-60, and K562) and antibacterial activity on six strains of pathogenic bacteria.

More details are available at the Supplementary Information.

## 3. Materials and Methods

### 3.1. General Experimental Procedures

Melting points were determined on an X-6 micromelting point apparatus and are uncorrected (Beijing CBIO Bioscience &Technologies Co., Ltd., Beijing, China). Optical rotations were measured on a JASCO P-1020 digital polarimeter (JASCO Corporation, Tokyo, Japan). IR spectra were recorded on a Nicolet-Nexus-470 spectrometer (International Equipment Trading Ltd., Vernon Hills, IL, USA) using KBr pellets. UV spectra were obtained on a Beckman DU 640 spectrophotometer (Beckman Coulter Inc., Brea, CA, USA). ECD spectrum was recorded on a JASCO J-810 circular dichroism spectrometer (JASCO Corporation, Tokyo, Japan). NMR spectra of compounds **1** and **3**–**5** were recorded on a JEOL JNM-ECP NMR spectrometer (JEOL Ltd., Tokyo, Japan; 600 MHz for ^1^H and 150 MHz for ^13^C), and NMR spectra of **2** and **6** were recorded on an Agilent DD2 500 MHz NMR spectrometer (Agilent Technologies, Inc., CA, USA; 500 MHz for ^1^H and 125 MHz for ^13^C). Chemical shifts (*δ*) were reported in ppm, using TMS as internal standard and coupling constants (*J*) were in Hz. ESIMS and HRESIMS were measured on a Micromass Q-TOF spectrometer (Thermo Fisher Scientific Inc., Waltham, MA, USA). HPLC separation was performed using a Hitachi prep-HPLC system coupled with a Hitachi L-2455 diode array detector. A Kromasil C_18_ preparative HPLC column (250 × 10 mm, 5 μm) was used (Hitachi Corporation, Tokyo, Japan). Silica gel (Qing Dao Hai Yang Chemical Group Co.; Qing Dao, China; 200–300 mesh), Sephadex LH-20 (Amersham Biosciences Inc., Piscataway, NJ, USA) and octadecylsilyl silica gel (Unicorn, Merck KGaA, Darmstadt, Germany; 45–60 μm) were used for column chromatography. Precoated silica gel plates (Yan Tai Zi Fu Chemical Group Co., Yan Tai, China; G60, F-254) were used for thin layer chromatography.

### 3.2. Materials

The gorgonian *Anthogorgia ochracea* GXWZ-07 (1.9 kg, wet weight) was collected from the coral reef of Weizhou Island in the South China Sea, China, in April 2011, and was identified by Dr. Xiu-Bao Li, South China Sea Institute of Oceanology, Chinese Academy of Science.

### 3.3. Extraction and Isolation

The gorgonian *A. ochracea* was cut into small pieces and exhaustively extracted with CH_2_Cl_2_/MeOH (*v*:*v*, 1:1) three times (3 × 2000 mL) at room temperature, and the solvent was evaporated *in vacuo*. The organic layer was filtered and concentrated under reduced pressure to give a residue (36.2 g), which was partitioned between EtOAc and H_2_O (*v*:*v*, 2:1) for three times. The EtOAc extract was concentrated *in vacuo* to afford 20 g of EtOAc residue, which was subjected to column chromatography (CC) on silica gel, using EtOAc-petroleum ether (0%–100%) as eluent. By combining the fractions with TLC monitoring, six fractions (Fr.1–Fr.6) were obtained. Fr.3 (1.2 g) was fractionated over silica gel CC eluted with EtOAc-petroleum ether gradients (10%–90%) to afford four sub-fractions (Fr.3.1–Fr.3.4). Repeated chromatography of Fr.3.3 using Sephadex LH-20 eluted with petroleum ether/CH_2_Cl_2_/MeOH (*v*:*v*:*v*, 2:1:1) provided Fr.3.3.1–Fr.3.3.3. Fr.3.3.1 was purified by ODS CC eluted with MeOH to yield **2** (4.5 mg), **3** (6 mg) and **6** (5.3 mg). Fr.3.3.2 was purified by semi-preparative HPLC (90% MeOH-H_2_O) to obtain **4** (4.3 mg). Fr.3.3.3 was purified by semi-preparative HPLC (60% MeOH-H_2_O) to obtain **1** (2.0 mg) and **5** (3.5 mg).

Ochracenoid A (**1**): red-purple solid; m.p. 299–301 °C; [α]^25^_D_ +30.7 (*c* 0.1, CH_2_Cl_2_); UV (MeOH) λ_max_ (log *ε*): 230 (3.91), 339 (3.89), 410 (3.36) nm; CD (0.10 mM, MeOH) λ_max_ (Δ*ε*) 278 (−0.96), 316 (+1.07), 384 (−0.73) and 432 (+0.52) nm; IR (KBr) ν_max_ 2337, 1655, 1530 and 1050 cm^–1^; ^1^H NMR (CDCl_3_, 600 MHz) and ^13^C NMR (CDCl_3_, 150 MHz), see Table 1; ESIMS *m*/*z* 241.1 [M + H]^+^; HRESIMS *m*/*z* [M + H]^+^ 241.1223 (calcd for C_16_H_17_O_2_, 241.1223).

Ochracenoid B (**2**): yellow solid; m.p. 202–204 °C; UV (MeOH) λ_max_(log *ε*): 206 (3.96), 248 (4.01), 330 (3.22) nm; IR (KBr) ν_max_ 1715, 1697, 1635, 1445 and 1210 cm^–1^; ^1^H NMR (CDCl_3_, 500 MHz) and ^13^C NMR (CDCl_3_, 125 MHz) see Table 2; ESIMS *m*/*z* 229.2 [M + H]^+^; HRESIMS *m*/*z* [M + H]^+^ 229.1223 (calcd for C_15_H_17_O_2_, 229.1223).

### 3.4. Biological Assays

The antiproliferative effects on zebrafish *Danio rerio* embryo were evaluated according to the described methods [25]. 3,4-Dichloroaniline was used as a positive control.

The cytotoxicities were evaluated for against human cervical carcinoma HeLa, human lung carcinoma A-549, and human colorectal cancer HCT-116 cell lines using SRB method [26] and human myeloid leukemia HL-60, and human leukemia K562 cell lines using MTT method [27]. Adriamycin was used as a positive control.

The antibacterial activities against six bacterial strains, *Staphylococcus epidermidis*, *S.* *aureus*, *Bacillus subtilis*, *B. cereus*, *Tetragenococcus halophilus*, and *Kocuria rhizophila*, were determined by a serial dilution technique using 96-well microtiter plates [28]. Ciprofloxacin was used as a positive control.

## 4. Conclusions

In summary, six guaiazulene-based analogues were obtained from a gorgonian *Anthogorgia ochracea* collected from the South China Sea. The planar structures of the new compounds (**1** and **2**) were elucidated by comprehensive spectroscopic data and the absolute configuration of **1** was determined by the comparison of TDDFT calculated electronic circular dichroism with its experimental spectrum. Compound **1** is a rare guaiazulene-based analogue possessing a unique C_16_ skeleton. A possible generation process of **1** through an intermolecular one-carbon-transfer reaction was also discussed. Compound **2** was previously described as a presumed intermediate involved in the biogenesis of anthogorgienes A and I. Compound **3** showed strong antiproliferative effects on the embryo development of zebrafish *Danio rerio*.

## Supplementary Files

Supplementary File 1 Supplementary Information (PDF, 767 KB)
