# Supplementary material for: Ochracenoids A and B, Guaiazulene-Based Analogues from Gorgonian Anthogorgia ochracea Collected from the South China Sea
Source: Mar Drugs. 2014 Mar 14;12(3):1569–79. doi: 10.3390/md12031569 (PMC3967227; doi:10.3390/md12031569)
Supplement: Supplementary File 1 — Supplementary Information (PDF, 767 KB) [file marinedrugs-12-01569-s001.pdf]

Supplementary Information

Table of Content

S1. 1D and 2D NMR and MS Spectra for Compounds 1 and 2
Figure S1. ^{1}H NMR spectrum (600 MHz, $CDCl_3$) of compound **1**.
Figure S2. ^{13}C NMR spectrum (150 MHz, $CDCl_3$) of compound **1**.
Figure S3. HMQC spectrum of compound **1** in $CDCl_3$.
Figure S4. HMBC spectrum of compound **1** in $CDCl_3$.
Figure S5. ^{1}H-^{1}H COSY spectrum of compound **1** in $CDCl_3$.
Figure S6. ESIMS spectrum of compound **1**.
Figure S7. HRESIMS spectrum of compound **1**.
Figure S8. ^{1}H NMR spectrum (500 MHz, $CDCl_3$) of compound **2**.
Figure S9. ^{13}C NMR spectrum (125 MHz, $CDCl_3$) of compound **2**.
Figure S10. HMQC spectrum of compound **2** in $CDCl_3$.
Figure S11. HMBC spectrum of compound **2** in $CDCl_3$.
Figure S12. ^{1}H-^{1}H COSY spectrum of compound **2** in $CDCl_3$.
Figure S13. ESIMS spectrum of compound **2**.
Figure S14. HRESIMS spectrum of compound **2**.
S2. ECD Simulation of Compound 1
Table S1. Calculation ECD data for the lowest energy conformer.

S1. 1D and 2D NMR and MS Spectra for Compounds 1 and 2

Figure S1. ^{1}H NMR spectrum (600 MHz, $CDCl_3$) of compound **1**.

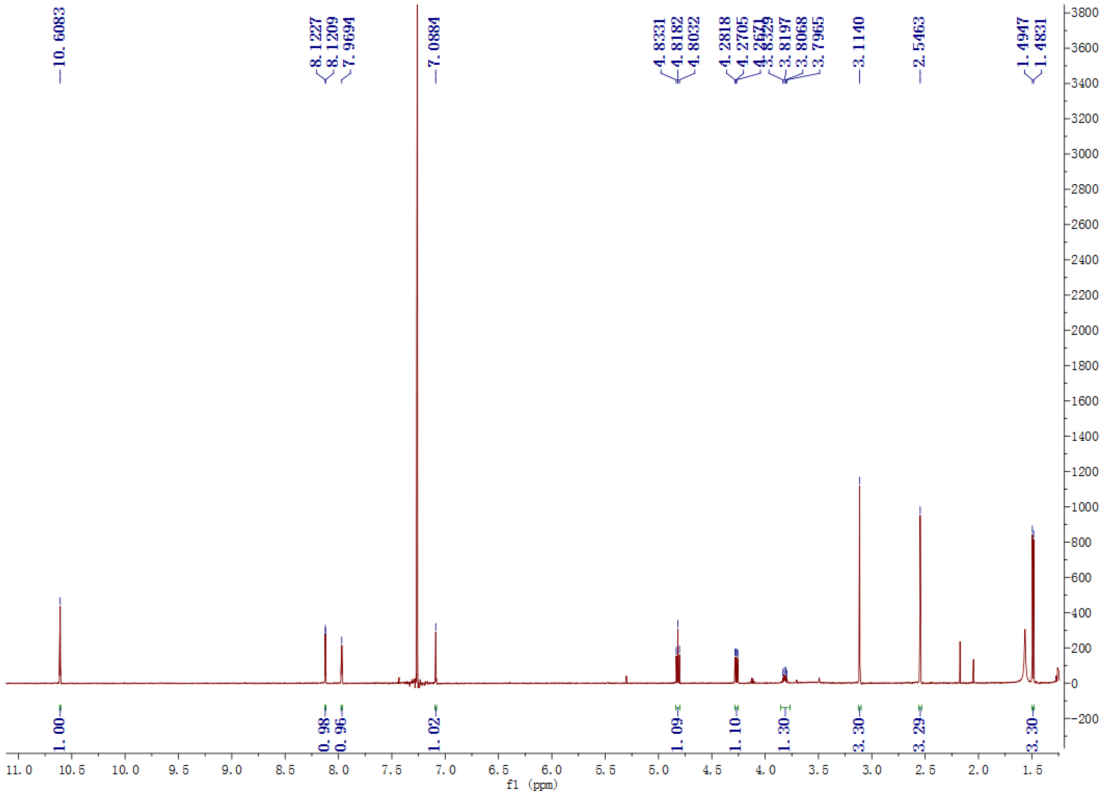

Figure S2. ^{13}C NMR spectrum (150 MHz, $CDCl_3$) of compound **1**.

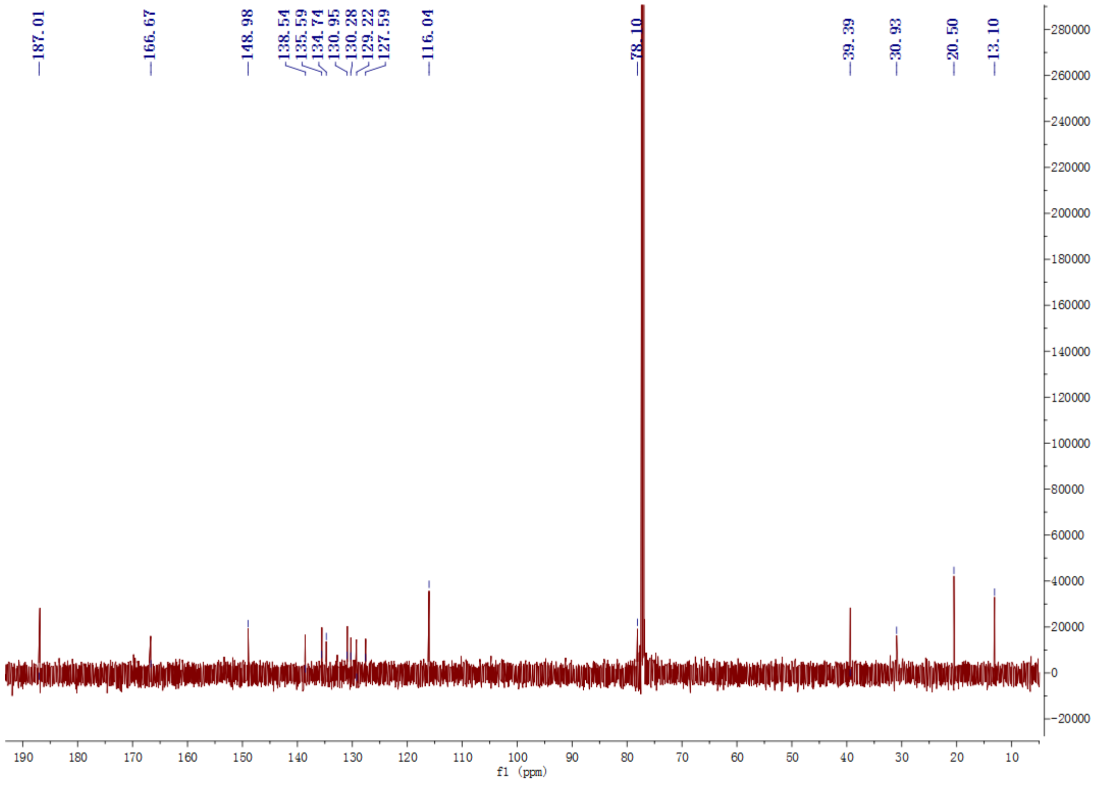

Figure S3. HMQC spectrum of compound **1** in $CDCl_3$.

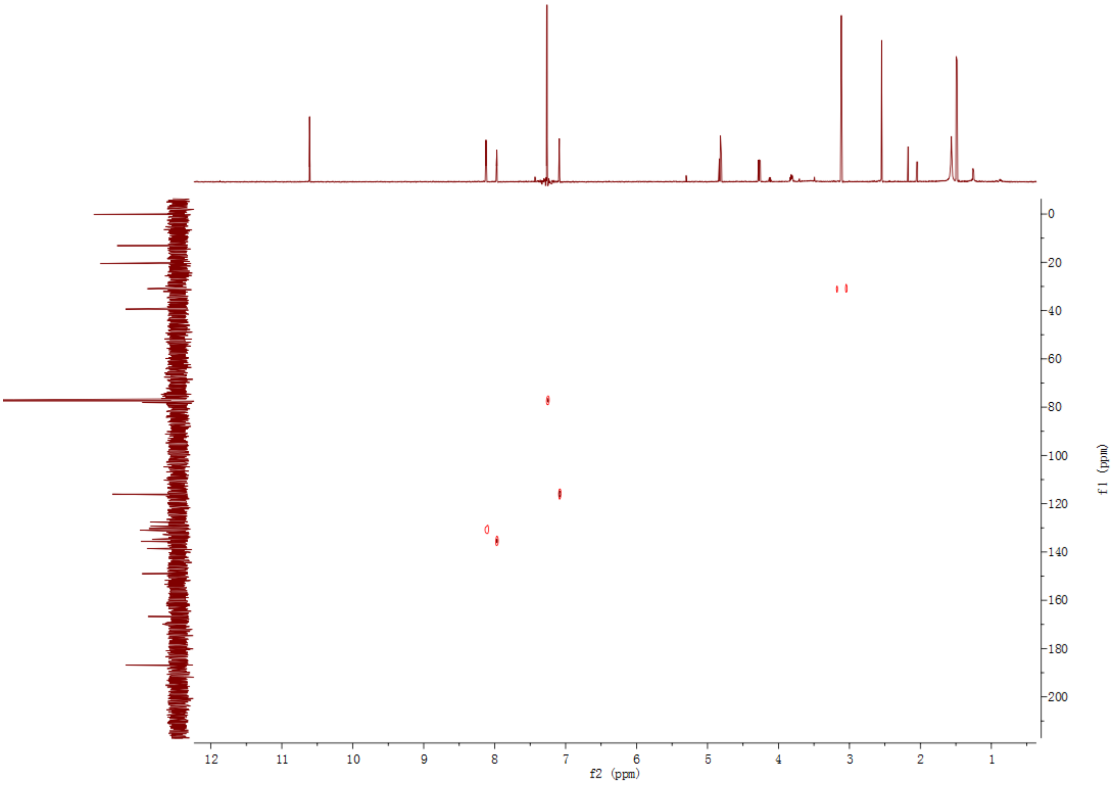

Figure S4. HMBC spectrum of compound **1** in $CDCl_3$.

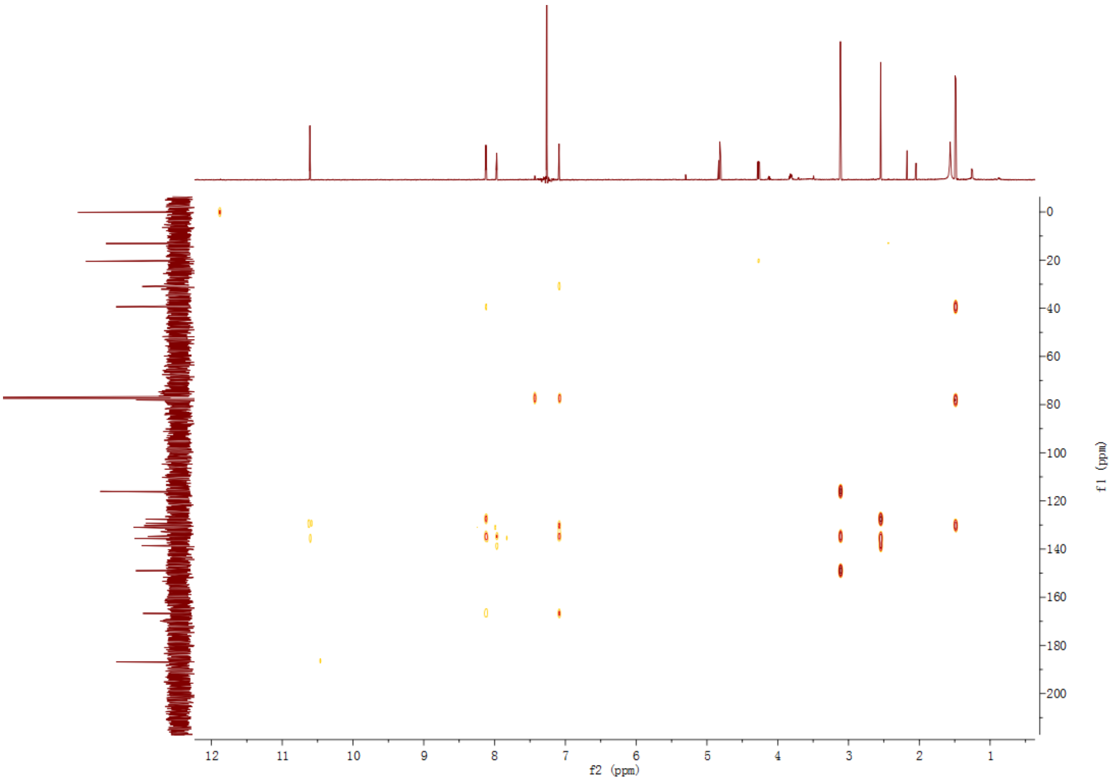

Figure S5. ^{1}H-^{1}H COSY spectrum of compound **1** in $CDCl_3$.

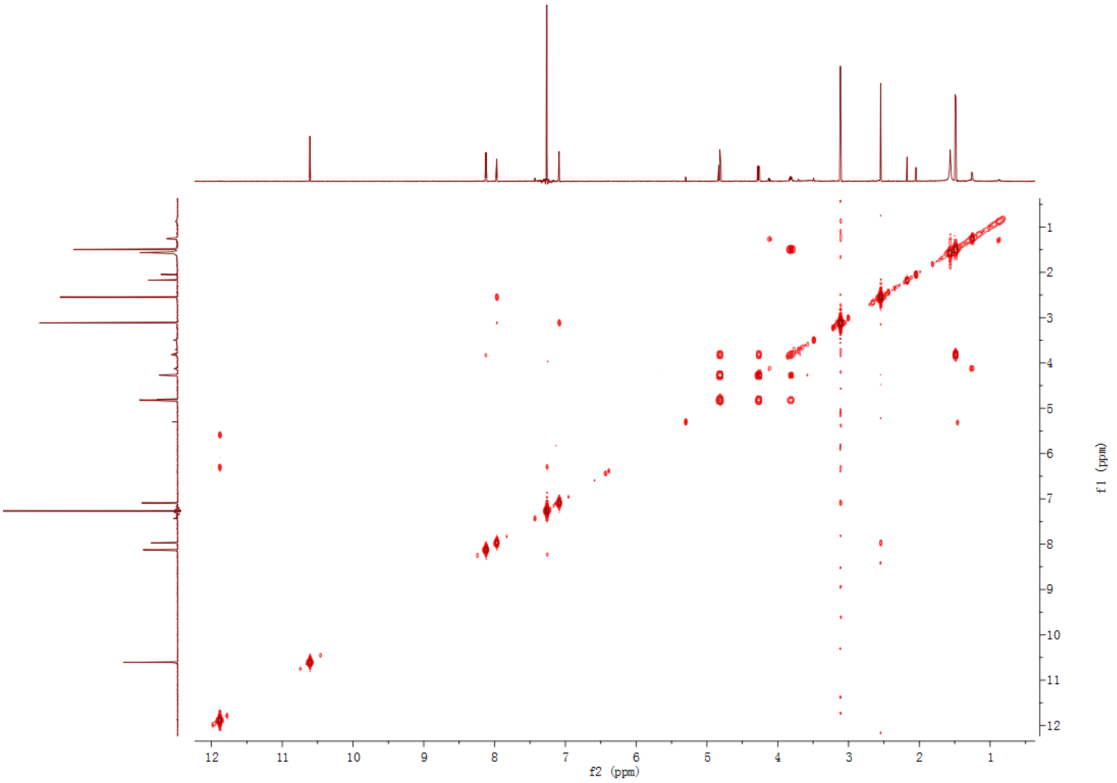

Figure S6. ESIMS spectrum of compound **1**.

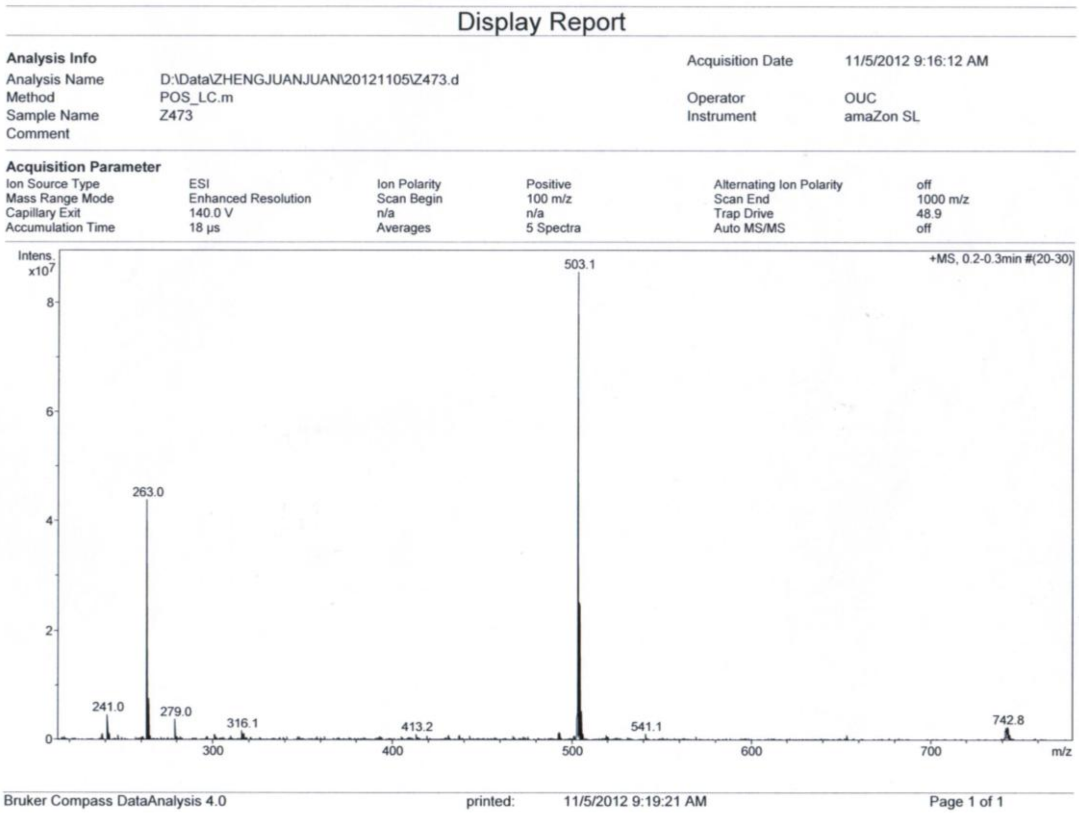

Figure S7. HRESIMS spectrum of compound **1**.

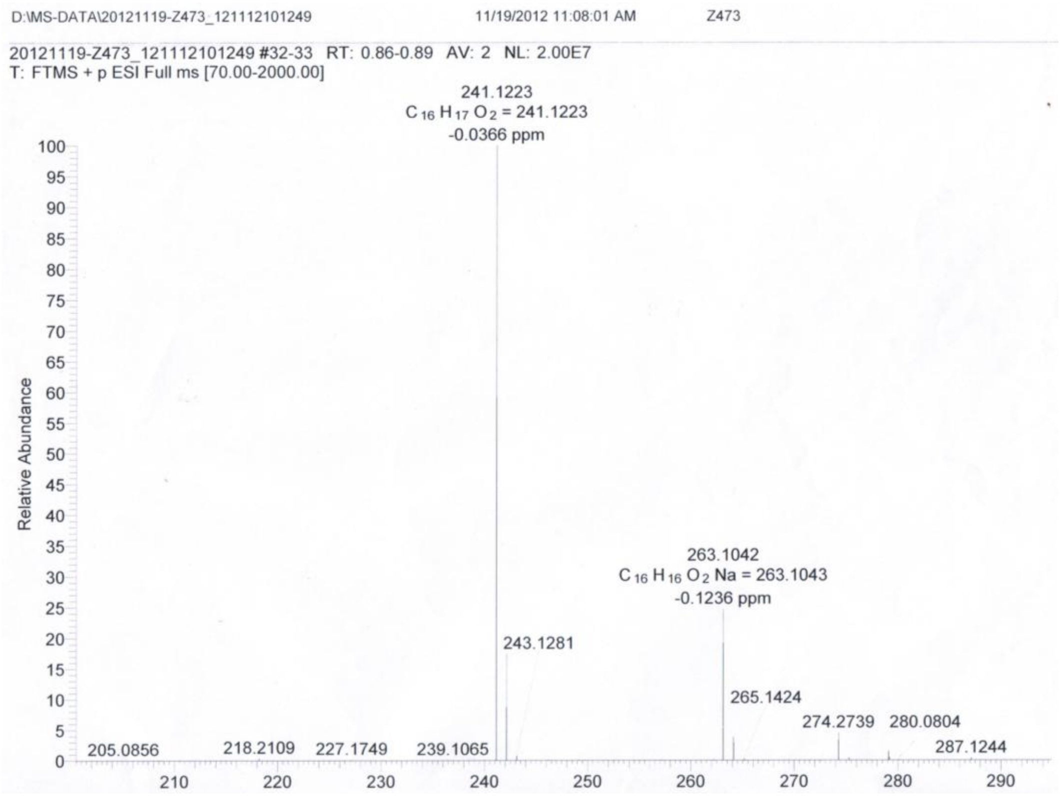

Figure S8. ^{1}H NMR spectrum (500 MHz, $CDCl_3$) of compound **2**.

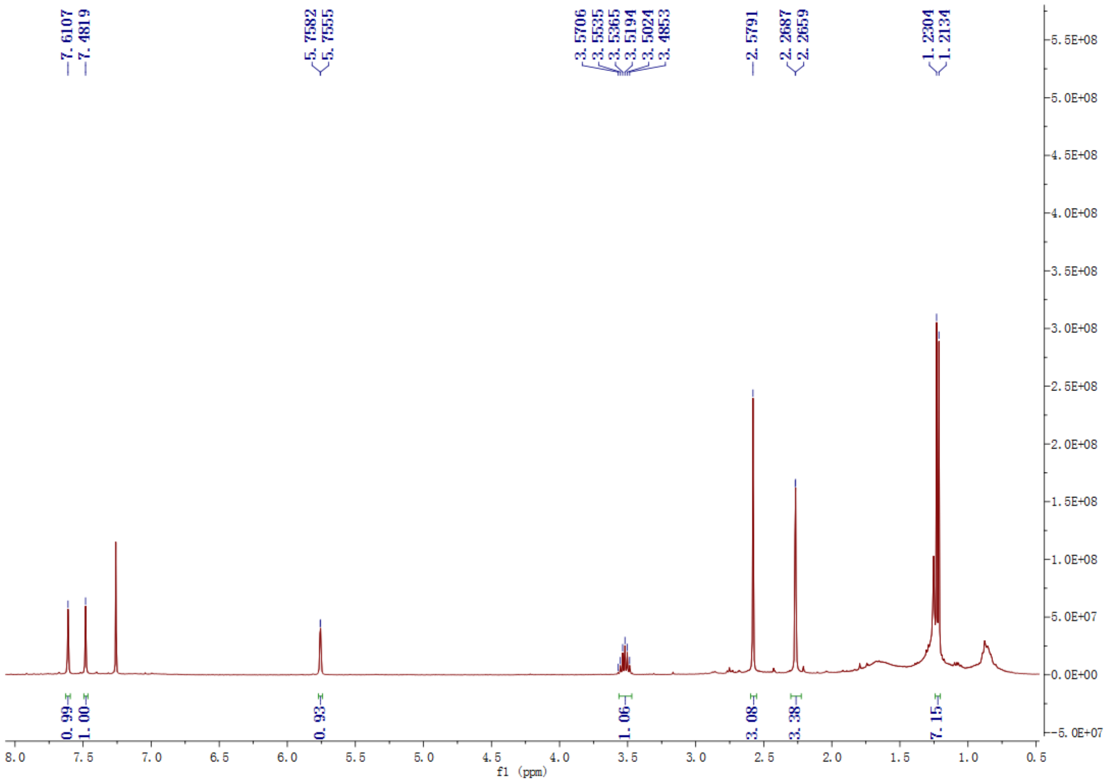

Figure S9. ^{13}C NMR spectrum (125 MHz, $CDCl_3$) of compound **2**.

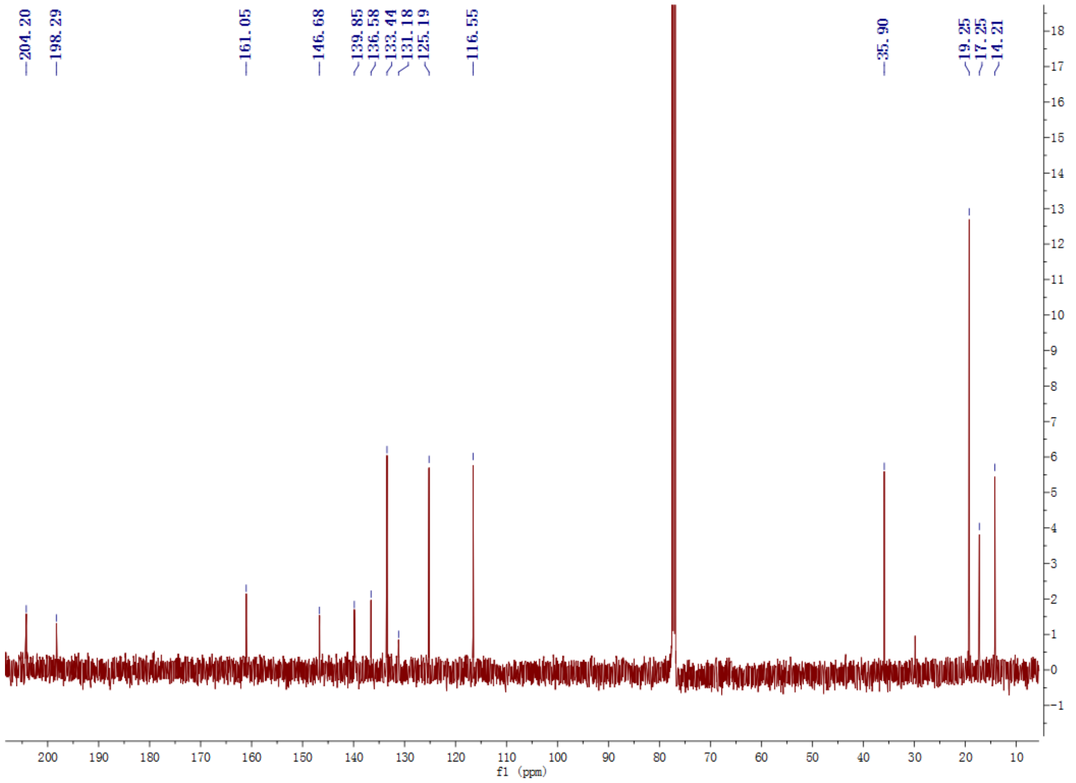

Figure S10. HMQC spectrum of compound **2** in $CDCl_3$.

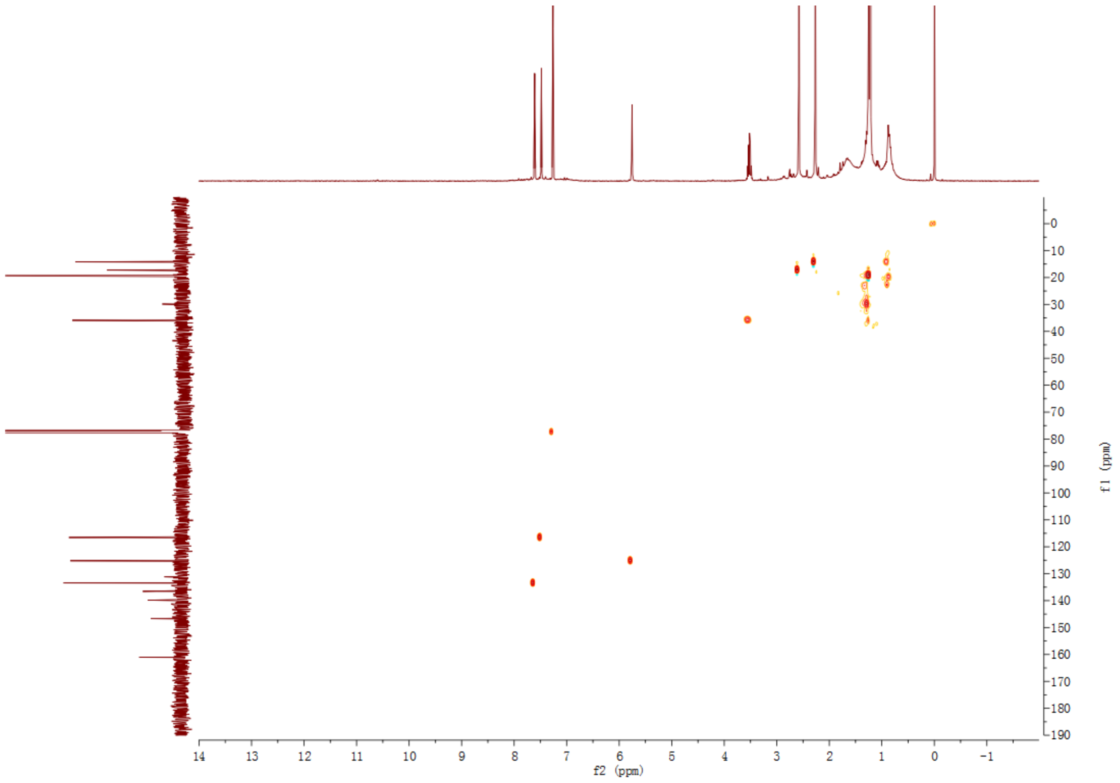

Figure S11. HMBC spectrum of compound **2** in $CDCl_3$.

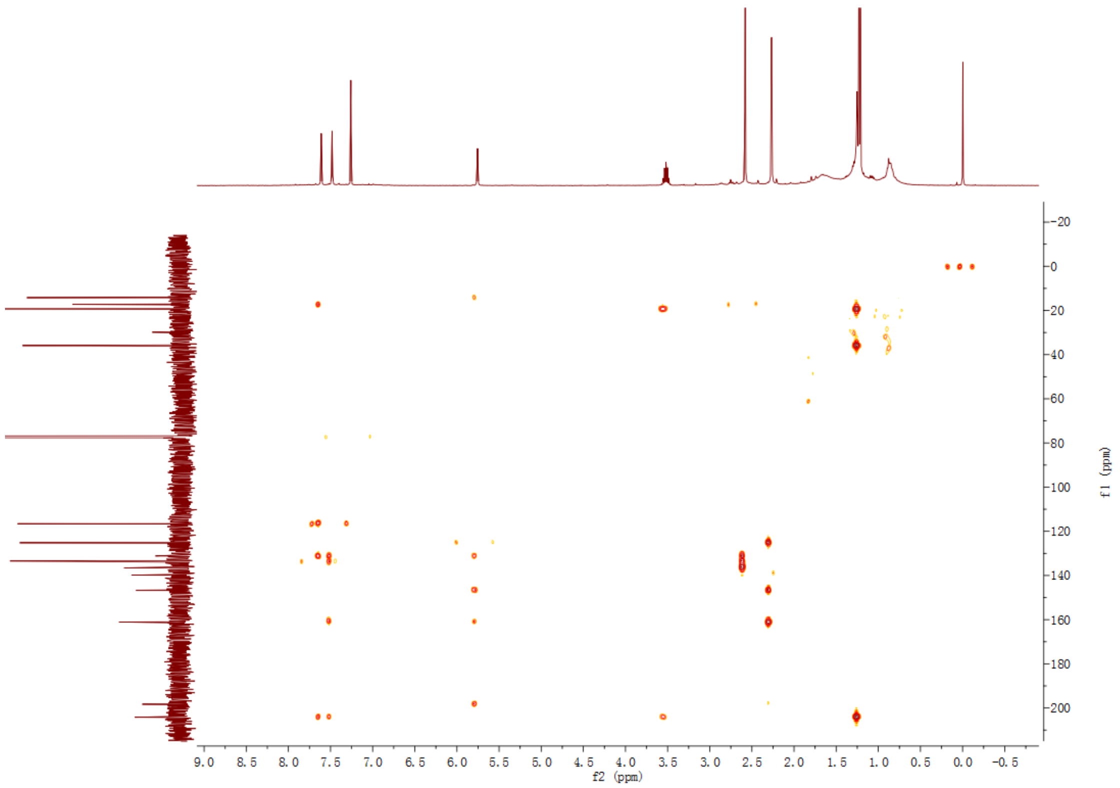

Figure S12. ^1H-^1H COSY spectrum of compound **2** in $CDCl_3$.

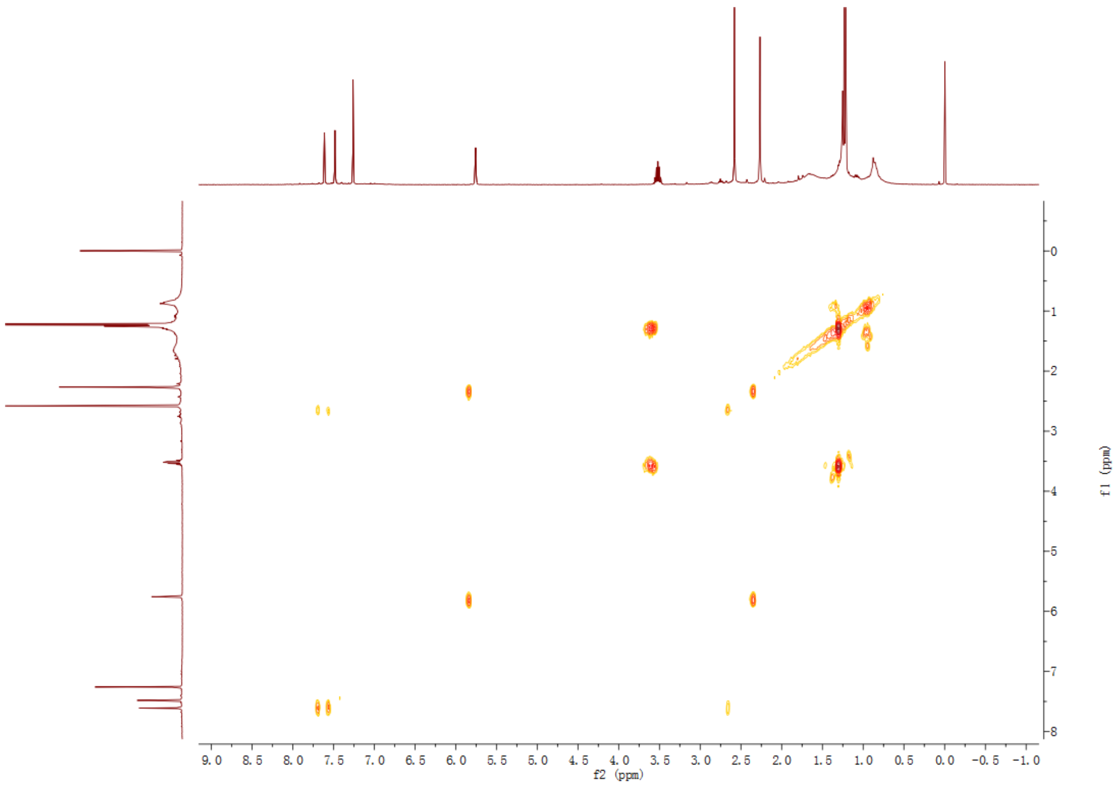

Figure S13. ESIMS spectrum of compound **2**.

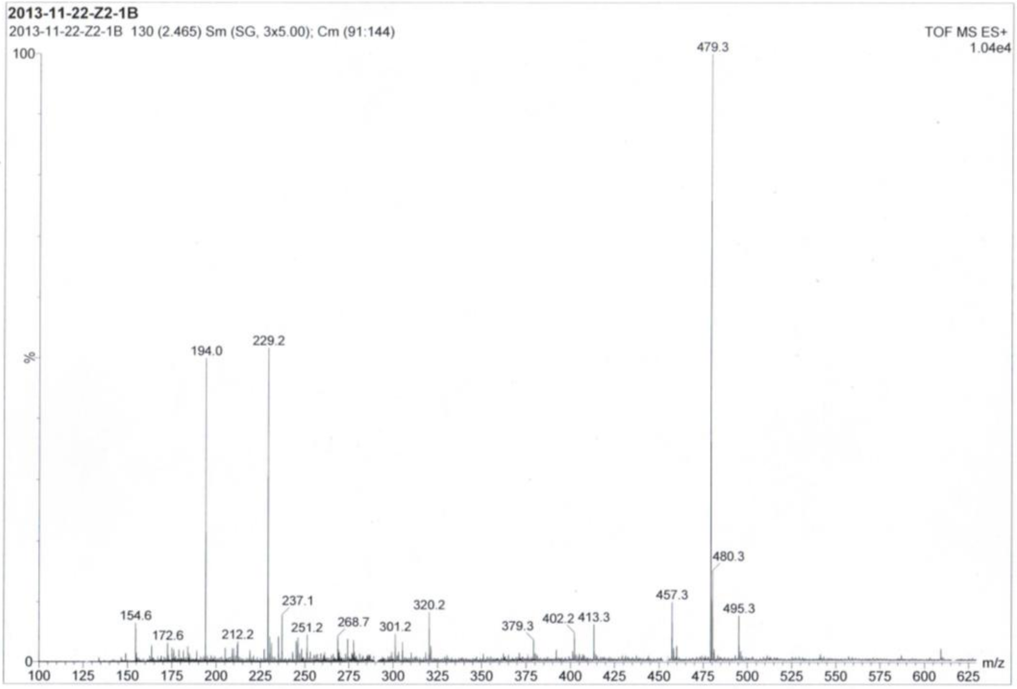

Figure S14. HRESIMS spectrum of compound **2**.

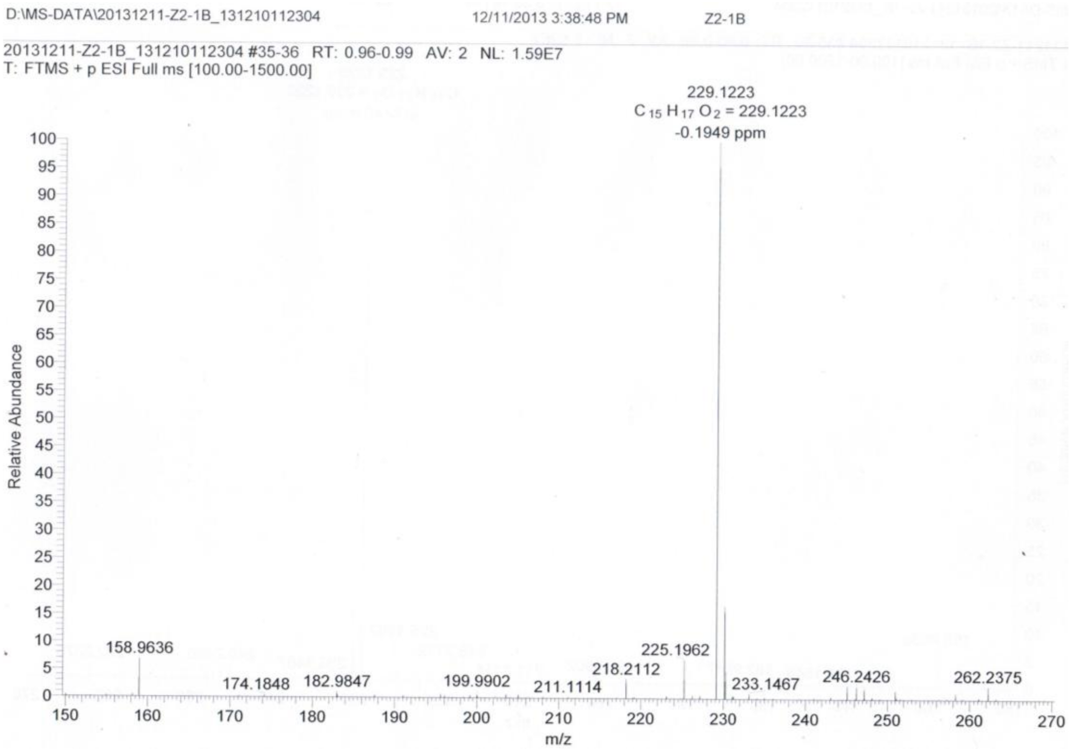

S2. ECD Simulation of Compound 1

ECD spectrum of each conformation is simulated according to the overlapping Gaussian functions expressed as:

$$\Delta\varepsilon(E) = \frac{1}{2.296\times10^{-39}\sqrt{\pi}\sigma}\sum_{i}^{A}\Delta E_i R_i e^{[-(E-\Delta E_i)^2/\sigma^2]}$$

where σ is half the bandwidth at 1/e peak height and expressed in energy units. The parameters ΔE_i and R_i are the excitation energies and rotational strengths for the transition *i*, respectively.

The above function is converted to Δε, λ (wavelength) correlations as:

$$\Delta\varepsilon(\lambda) = \frac{1}{2.296\times10^{-39}\sqrt{\pi}\sigma}\sum_{i}^{A}\Delta E_i R_i e^{[-(1240/\lambda-\Delta E_i)^2/\sigma^2]}$$

and then simulation were accomplished by using the Excel 2003 and the Origin 7.0 software. Velocity rotatory strengths were used for ECD curve simulation.

Table S1. Calculated ECD data for the lowest energy conformer.

State	Excitation Energies (eV)	Wavelength (nm)	Rotatory Strengths *	State	Excitation Energies (eV)	Wavelength (nm)	Rotatory Strengths *
1	2.5249	491.04	0.9032	16	5.374	230.71	0.9463
2	3.1719	390.88	−0.3886	17	5.4449	227.71	11.6716
3	3.3081	374.79	−2.009	18	5.5202	224.6	−2.0132
4	3.6536	339.35	−3.9165	19	5.5364	223.94	−12.1664
5	3.6575	338.99	−2.3367	20	5.5656	222.77	1.2979
6	4.1002	302.39	20.9464	21	5.5783	222.26	0.9404
7	4.5062	275.14	1.7038	22	5.6291	220.25	−0.1661
8	4.9127	252.37	3.6723	23	5.6671	218.78	4.7842
9	4.9506	250.44	−16.3335	24	5.7234	216.63	15.7885
10	4.9968	248.13	−20.8979	25	5.8068	213.52	−4.8249
11	5.0516	245.43	0.4355	26	5.8404	212.29	0.1877
12	5.0734	244.38	2.4068	27	5.8575	211.67	4.9851
13	5.192	238.8	7.1343	28	5.8918	210.43	16.7355
14	5.2298	237.07	9.4053	29	5.9492	208.4	3.1227
15	5.3204	233.04	−7.2738	30	6.0034	206.52	0.3082

* *R*(velocity) 10**-40 erg-esu-cm.

© 2014 by the authors; licensee MDPI, Basel, Switzerland. This article is an open access article distributed under the terms and conditions of the Creative Commons Attribution license (http://creativecommons.org/licenses/by/3.0/).
